# Deep Eutectic Solvents (DESs) and Their Application in Biosensor Development

**DOI:** 10.3390/s21134263

**Published:** 2021-06-22

**Authors:** Rossella Svigelj, Nicolò Dossi, Cristian Grazioli, Rosanna Toniolo

**Affiliations:** Department of Agrifood, Environmental and Animal Science, University of Udine, Via Cotonificio 108, 33100 Udine, Italy; nicolo.dossi@uniud.it (N.D.); cristian.grazioli@uniud.it (C.G.)

**Keywords:** deep eutectic solvents, biosensors, DNA, enzymes, MIPs, aptamers, nanomaterials

## Abstract

Deep Eutectic Solvents (DESs) are a new class of solvents characterized by a remarkable decrease in melting point compared to those of the starting components. The eutectic mixtures can be simply prepared by mixing a Hydrogen Bond Acceptor (HBA) with a Hydrogen Bond Donor (HBD) at a temperature of about 80 °C. They have found applications in different research fields; for instance, they have been employed in organic synthesis, electrochemistry, and bio-catalysis, showing improved biodegradability and lower toxicity compared to other solvents. Herein, we review the use of DESs in biosensor development. We consider the emerging interest in different fields of this green class of solvents and the possibility of their use for the improvement of biosensor performance. We point out some promising examples of approaches for the assembly of biosensors exploiting their compelling characteristics. Furthermore, the extensive ability of DESs to solubilize a wide range of molecules provides the possibility to set up new devices, even for analytes that are usually insoluble and difficult to quantify.

## 1. Introduction

### 1.1. Deep Eutectic Solvents (DESs)

Lately, a new class of solvents, called Deep Eutectic Solvents (DESs), has been successfully employed in different research fields. In 2004, Abbott et al. described the generation of eutectic mixtures as complexes formed between halide salts and a range of compounds such as amides and carboxylic acids [1]. Indeed, DESs can be easily prepared by mixing at least two components able to form a eutectic mixture with a melting point lower than that of the starting components [2,3]. This group of solvents share some characteristics with ionic liquids (ILs), a class of organic salts with a low melting point, generally obtained by combining an organic cation (commonly imidazolium-based cations) with a large variety of anions (i.e., Cl^−^, BF_4_^−^, PF_6_^−^, [NTf_2_]^−^) [4]. However, DESs have demonstrated to have some interesting advantages over ILs. As a matter of fact, the definition of ILs as “green” species is largely contested in the current literature [5]. Therefore, to overcome the high cost of their production and their toxicity, DESs have arisen as a cheaper and greener alternative [6].

Recently, another class of solvents, called Natural Deep Eutectic Solvents (NADESs), has been introduced [7]. This new category was described after observing the occurrence of certain natural mixtures. In fact, the presence of eutectic liquids in living organisms could explain a great number of biological processes where poorly water-soluble metabolites are involved [8]. In different types of microbial, animal, and plant cells, the presence of substances, including sugars, some amino acids, choline, and some organic acids such as malic acid, citric acid, and lactic acid, could lead to the formation of NADESs as a third type of solvent besides water and lipids. Furthermore, the existence of these natural solvents could explain the survival of some organisms through extreme conditions such as cold or water shortage [8].

**Figure 1:**
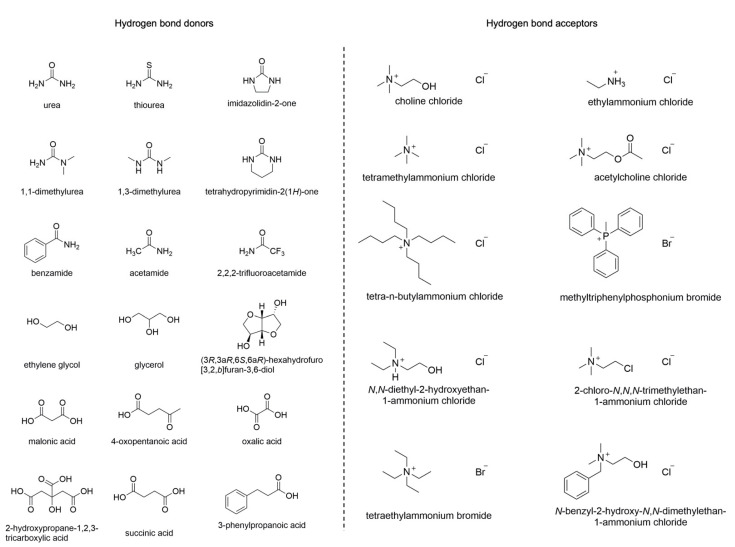
Typical HBDs and HBAs employed in DESs.

DESs are usually prepared by mixing a Hydrogen Bond Acceptor (HBA) with a Hydrogen Bond Donor (HBD) at a temperature of about 80 °C. Among the HBAs, different quaternary ammonium salts can be found, while amines, carboxylic acids, alcohols, or carbohydrates belong to the group of HBDs (Figure 1). DESs have found applications in various research areas, including organic synthesis, electrochemistry, and bio-catalysis [9]. Furthermore, they display improved biodegradability and lower toxicity compared to other solvents, characteristics that make them particularly interesting in green chemistry [10].

Interestingly, both DESs and NADESs provide a network of hydrogen bonds that make possible the solubilization of a wide range of molecules [11]. Their characteristics make them potential universal solvents capable of extracting a wide variety of non-polar and polar compounds [7]. Thus, they could become an appealing alternative to several standard and toxic organic solvents. The structures of their components strongly influence all DESs’ physicochemical properties, such as melting point, density, conductivity, and viscosity [12]. Moreover, studies on the three-dimensional structure of DESs revealed that the interaction between HBDs and HBAs depends mainly on hydrogen bonds, but they also interact with the anion by arranging themselves around it [13]. In general, the presence of a broad hydrogen-bonding network among the components is responsible for the high viscosity of DESs and the restrained mobility of free species inside the solvent. Additional phenomena, such as van der Waals and electrostatic interactions, may also be responsible for the high viscosity of DESs. The freezing point of DESs is characterized by a consistent drop, generally wider than 150 °C. The difference in the freezing point of a binary mixture in the eutectic composition compared to that of a theoretical ideal mixture is related to the magnitude of the interaction of the components [10]. DESs exhibit, in general, higher densities than water, with values ranging from 1.041 g cm^−3^ to 1.63 g cm^−3^ [10]. Among the different quaternary ammonium salts used in eutectic mixtures, choline chloride (ChCl) is the most employed thanks to its low cost and biodegradability. ChCl can be combined with HBDs to provide DESs with different physical and chemical properties such as freezing point, viscosity, conductivity, and pH [14]. Table 1 shows some physical parameters of ChCl-based DESs.

To date, DESs have found many applications in analytical chemistry, such as in the extraction of analytes from complex liquid and solid matrices [15], as modification media for nanomaterials [16,17], for elution in dispersive solid-phase extractions, and as a mobile-phase modifier in chromatography [18,19]. They have also been employed in the extraction of bioactive compounds [20], including flavonoids, phenolic acids, polyphenols, saponins, and anthraquinones, from various types of natural sources [21,22,23]. In addition, DESs display a good capability of solubilizing many other compounds, such as drugs, metal oxides, and carbon dioxide [24,25].

Taking into account all their properties and their biocompatibility, this class of solvents could replace the classic aqueous buffers that are normally used in the development of biosensors. DESs could also improve biosensor performance, for example, in the analysis of molecules that are not soluble in water.

**Figure 2:**
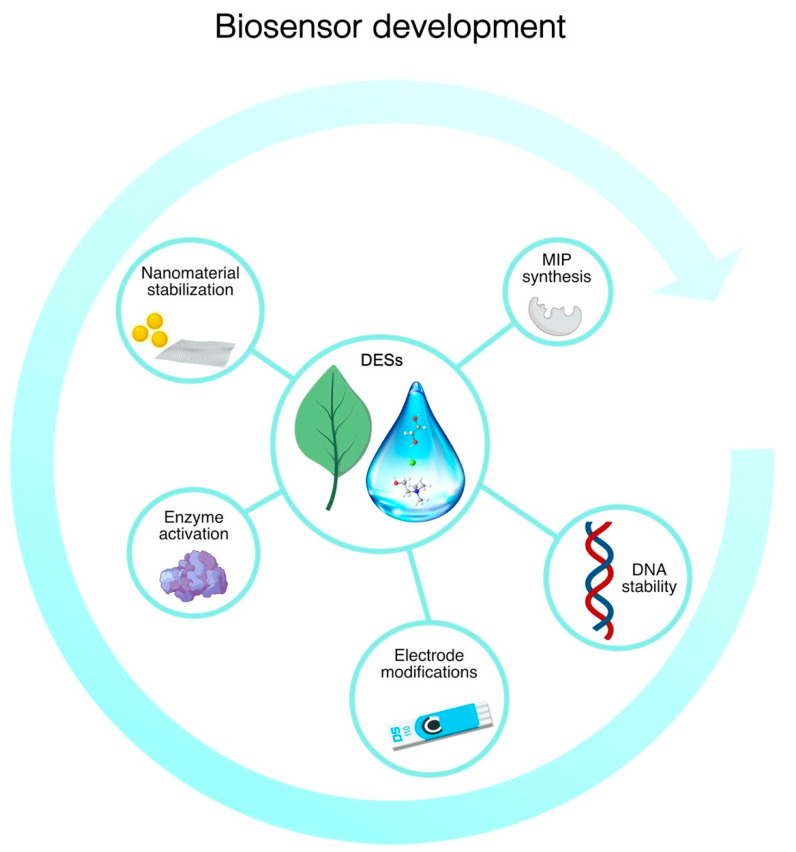
Overview of the general advantages of the use of DESs in biosensor development. The use of DESs can improve the performance of biosensors thanks to the biocompatibility of this class of solvents with the recognition elements and the improvement of electrode material performance.

Here we discuss the recent uses of DESs in biosensor development and their combination with different materials to enhance the sensing performance of biobased sensing devices, underlining the advantages that these solvents bring to the construction of miniaturized and green biosensors (Figure 2). DESs have been used for the generation of nanomaterials and the modification of electrodes with graphene, carbon paste, and carbon nanotubes. Furthermore, DESs stabilize DNA molecules, favoring the development of stable biosensors; they increase the activity of some enzymes, providing better sensitivity; and finally, they can favor the creation of MIPs for poorly soluble molecules.

### 1.2. Biosensors

**Figure 3:**
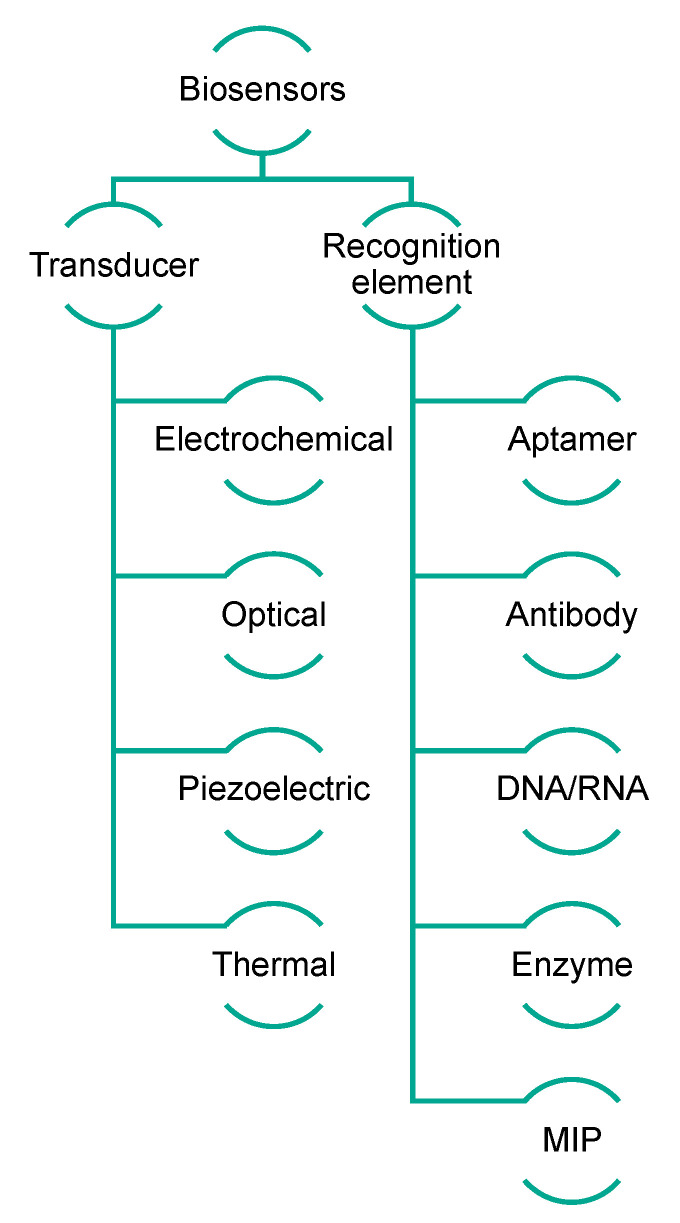
Schematic representation of biosensor classification.

Biosensors exploit the sensitivity of transducers combined with the high specificity of biological recognition elements, which are able to interact selectively with analytes [26]. Generally, biosensors can be categorized on the basis of their transduction mechanism, which can be optical (including optical fiber and surface plasmon resonance biosensors), electrochemical (including voltammetric, amperometric, and impedance biosensors), or piezoelectric (including quartz crystal microbalance biosensors) [27]. Alternatively, their classification is based on the sensing element, in which case they are named immunosensors, aptasensors, genosensors, enzymatic biosensors, and molecularly imprinted sensors when the biological sensing elements are antibodies, aptamers, nucleic acids, enzymes, and MIPs (molecularly imprinted polymers), respectively (Figure 3) [28,29]. The impact of biosensing is gaining importance in all sectors, such as clinical, environmental, and food-related fields [30,31]. Biosensors can be considered very versatile and powerful tools because of their low cost and compatibility with portable and compact instrumentation, as well as their easy and rapid use.

Electrochemical biosensing platforms arise from the combination of different disciplines such as chemistry, material science, biology, and electronic engineering. A significant example of successful electrochemical biosensing is the glucose sensor that relies on the interaction of the analyte with an enzyme (glucose oxidase), giving rise to a current directly proportional to the blood glucose level [32]. In the last years, great progress has been made: for instance, a continuous glucose monitoring system has been introduced that allows better control of a patient’s blood glucose levels [33]. This technology is based on implantable transmitters detecting blood sugar levels directly in the body. With the same purpose, wearable electrochemical sensors have been developed which integrate, in a non-invasive and non-obtrusive way, sensing systems suitable for various monitoring applications directly onto either textile materials or the patient epidermis [33,34].

All these innovations show that it is possible to develop technologies that can have real repercussions on the market and that the demand for advanced portable and green devices will extensively grow in the near future.

## 2. Synthesis of Electrode Materials in DESs

The use of DESs can improve the performance of electrode materials. Recently, some works have reported the application of DESs in the development and use of carbon paste electrodes and of solid electrodes modified with graphene, nanoparticles, and carbon nanotubes. Within the development of different materials, DESs can offer several advantages, including avoiding the aggregation of nanoparticles and allowing an effective functionalization of nanotubes. Moreover, the high solubility of various types of chemicals, like metal salts, metal oxides, solid halogens, and small organic molecules, and green characteristics are attractive features associated with the use of DESs and explain their increasing use.

### 2.1. Graphene, Carbon Paste, and Carbon Nanotubes in DESs

Several materials have been modified with DESs, including graphene, carbon paste, and nanotubes. The modification of these materials with the eutectic mixtures has shown several advantages, including increased charge transfer and conductivity, increased surface area, and the generation of new functional groups.

Graphene and DESs have separately demonstrated encouraging properties in the development of electroanalytical platforms [35]. Recently, Fuchs et al. combined the use of graphene and DESs, studying their coadjuvant effects in electrochemistry [36]. They considered the electrochemical performance of a centimeter-scale graphene monolayer generated by chemical vapor deposition and afterward moved onto insulating SiO_2_/Si supports in the DES ethaline formed by 1:2 ChCl and ethylene glycol. They provided a first approach for the use of graphene combined with a DES for electroanalytical applications, covering also the characterization of the graphene/DES electrochemical behavior. The authors also investigated the modification of graphene with metal (Zn) and metalloid (Ge) in the DES, evaluating their electrochemical performances. What emerged was that the behavior of graphene in ethaline is close to the characteristics of glassy carbon or highly ordered pyrolytic graphite; however, other features of the graphene/ethaline systems are uncommon, thanks to the two-dimensional nature of graphene. Hence, this work gave the first framework for graphene–DES systems.

A recent work by Cariati and Buoro reported the employment of natural deep eutectic solvents (NADESs) for the modification of carbon paste electrodes [37]. Enhanced conductivity and charge transfer rate were noticed when the cyclic voltammograms of potassium ferrocyanide were recorded with the NADES-modified carbon paste electrode compared to the bare carbon paste, revealing smaller peak potential separation. An improvement in the diffusion of the probe to the graphite surface was achieved with the integration of KCl in the NADES solution, decreasing in this way the binder layer viscosity. Better performance when using a reline (ChCl and urea, 1:2) modified carbon paste electrode was also noted for the oxidation of dopamine and ascorbic acid.

Further, Ibrahim et al. prepared two DESs using ethylene glycol combined with *N*,*N*-diethylethanolammonium chloride, or ChCl. In this study, the capability of DESs for carbon nanotube functionalization was investigated by studying the changes occurring after the functionalization process, using KMnO_4_ as the oxidizing agent. After the modifications, the functionalized carbon nanotubes were tested for the absorption of methyl orange from water. The effect of DESs was an increased surface area of carbon nanotubes and the generation of new functional groups on the carbon nanotubes’ surfaces without causing any damage to their structure, demonstrating its usefulness in the development of modified carbo-nanotubes for water treatment [38]. Furthermore, Rozas et al. studied the properties of carbon, boron nitride, silicon, germanium, and molybdenum disulfide nanotubes in reline by applying classical molecular dynamics simulations [39]. In this study, the interactions between reline and nanotubes revealed the development of a strongly adsorbed layer, demonstrating a great affinity of reline for the considered nanotubes and, among them, especially for the boron nitride one. The authors described a sustainable DES-based nanofluid system applicable to many different research fields. The nanotube size effect was also studied and, in every case, reline was able to solvate the nanotubes, demonstrating that nanofluids based on reline can be synthesized for a wide range of nanotube sizes and chemical compositions for different nanotechnology applications.

### 2.2. Nano and Magnetic Particles in DESs

Nanoparticles and magnetic particles have proved their importance in the development of different analytical platforms. Although they present several advantages, among the disadvantages are their aggregation and lack of stability over time; regarding these aspects, the use of eutectic liquids could help expand their use.

In 2008, Liao et al. reported the synthesis of gold nanoparticles (AuNPs) with diverse and unique shapes such as stars, snowflakes, and thorns using reline [40]. The authors obtained star-shaped AuNPs using HAuCl_4_ and l-ascorbic acid in DES at room temperature. They outlined that the water content present in the DES can modulate different shapes. SEM images illustrated pentagonal star-shaped AuNPs of about 300 nm; the images also revealed the presence of three- and four-branch star-shaped nanoparticles. The fact that DESs can influence the shape of nanoparticles could lead to progress in the synthetic approaches for nanomaterials.

In 2014, another synthesis and growth mechanism of AuNPs in DES was described [41]. In this study, a low-energy, soft-sputtering deposition technique was employed for gold nanoparticle formation in DES. The authors reported that DES can act as a structure-directing agent in the self-assembly (SA) of gold nanoparticles, and this feature can be modulated by anions, cations, and neutral components, such as urea. In addition, high concentrations of AuNPs in the eutectic mixture resulted in SA in the first and second shell short-range ordering of the gold nanoparticles. The growth and the ordering of AuNPs occurred on the surface as well as inside the bulk DES. The SA process depended on the nature of the components forming the eutectic mixture, and the authors employed reline, which is based on ChCl and urea. They obtained AuNPs of 5 nm size and SA in short-range order when they employed reline, while when using a non-templating agent such as castor oil they obtained AuNPs but without the SA. Moreover, the use of DES ensured that the particles remained stable, showing no aging effect, demonstrating the stabilizing effect of DES.

Svigelj et al. monitored the behavior of magnetic particles (MPs) modified with the peptide 33-mer in buffer and in ethaline with backscattering experiments. The MPs dispersed in ethaline remained in suspension longer than those in buffer, where they precipitated (speed of 0.06 mm/h) after undergoing agglomeration, which was detected as an increase in the backscattering of light in the middle zone of the measurement cell. In the DES, the particles displayed a homogeneous distribution throughout the entire cell and remained stable for 24 h, with no precipitation [42]. Among the advantages of the use of DES combined with MPs, the authors highlighted the stabilization and the ease of manipulation.

**Figure 4:**
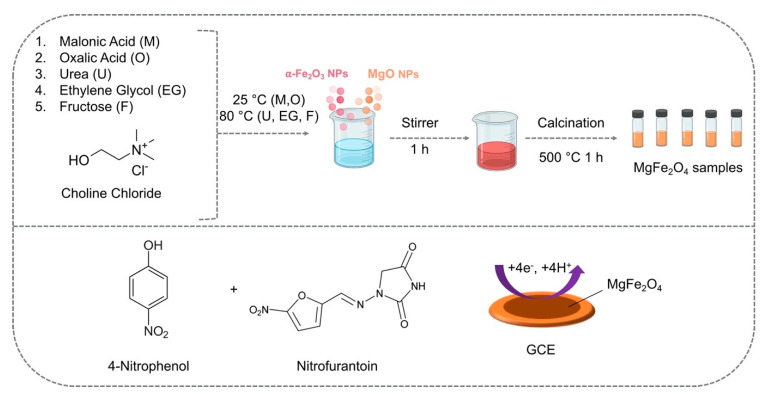
Synthetic path of various DES-assisted MgFe_2_O_4_ and their use in the electrochemical detection of 4-NP and NFT. Reprinted (adapted) with permission from Baby, J.N.; Sriram, B.; Wang, S.-F.; George, M. Effect of Various Deep Eutectic Solvents on the Sustainable Synthesis of MgFe_2_ O_4_ Nanoparticles for Simultaneous Electrochemical Determination of Nitrofurantoin and 4-Nitrophenol. *ACS Sustain. Chem. Eng.* **2020**, *8*, 1479–1486. Copyright 2020, American Chemical Society [43].

More recently, Baby et al. employed DESs in the solid-state synthesis of phase-pure magnesium ferrite nanoparticles that were subsequently exploited for the concomitant detection of nitrofurantoin (NFT) and 4-nitrophenol (4-NP) [43]. Five different DESs, based on the combination of ChCl with malonic acid, oxalic acid, urea, ethylene glycol, and fructose, were employed in the synthesis of the magnesium ferrite nanoparticles (Figure 4).

The authors noted that the type of DES employed in the synthesis was decisive for the shape of the NPs. The use of DESs based on ChCl and malonic acid or oxalic acid provided NPs with a variety of cubical and spherical shapes, whilst NPs synthesized in the DES based on ChCl and urea consisted of cubical nanoparticles. When ChCl in combination with ethylene glycol was used, nanospheres and spherical agglomerations were obtained, while the use of a DES based on ChCl and fructose led to the formation of nanograins. The electrode modified with NPs synthesized in the ChCl and fructose DES showed the highest sensitivity and lowest detection limits and a linear range between 0 and 342.6 μM, showing also an excellent selectivity and a good reproducibility.

### 2.3. Synthesis of Molecularly Imprinted Polymers in DESs

Usually, molecularly imprinted polymers (MIPs) are developed against a specific target that is used as template; however, some target compounds are not suitable as templates because of their poor solubility in common solvents. The tailoring properties of DESs could overcome this issue, allowing the production of MIPs for a wider range of molecules.

In this regard, Fu et al. obtained an MIP for caffeic acid using a ternary ChCl–caffeic acid–ethylene glycol (ChCl–CA–EG) DES, which was employed as a template to prepare MIPs. The specific recognition capacity of the MIPs for polyphenols was better than those of C18, C8, or non-molecularly imprinted polymer adsorbents, and was favorably employed for the determination of polyphenols extracted from a sample of *Radix asteris* [44]. The authors proved that transformation of an insoluble target compound in a polymeric DES for MIP preparation and recognition is an innovative and viable strategy for further MIP developments.

**Figure 5:**
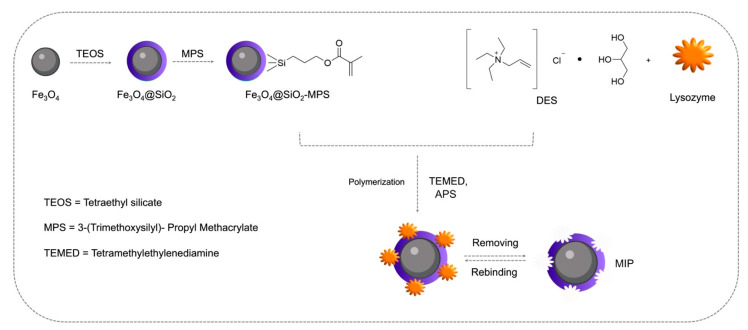
Schematic illustration of the preparation of DES-Fe_3_O_4_@SiO_2_-MIP. Adapted with permission from [45].

The use of DESs in the synthesis of MIPs is a promising approach for specific recognition of proteins. As a matter of fact, the imprinting for biomacromolecules, like proteins, represents a difficult goal to achieve because of their molecular size, low mass transfer, and complex and changeable conformation. A step towards the solution of these problems has been taken with the introduction of DESs in the preparation of MIPs. Xu et al. designed and synthesized a DES-based magnetic surface imprinted polymer for the selective recognition of lysozyme [45]. The obtained DES-Fe_3_O_4_@SiO_2_-MIP displayed better adsorption ability for the template lysozyme than DES-Fe_3_O_4_@SiO_2_-NIP (non-imprinted polymer). Adsorption experiments using the single reference protein, a binary protein mixture, and, finally, a real sample were performed to prove the selectivity and specificity of the imprinted polymer. The authors demonstrated that recognition holes are generated on the surface of DES-Fe_3_O_4_@SiO_2_-MIP during the imprinting process (Figure 5). They also proved the reusability of the imprinted polymer, which can be regenerated with good stability.

Li et al. employed four types of DESs for the modification of magnetic MIPs with multiple templates based on silica. Subsequently, the authors applied the imprinted polymers for the fast and concomitant magnetic solid-phase extraction of active diterpenoids from *Salvia miltiorrhiza bunge*, major isoflavones from *Glycine max (Linn.) Merr*, and catechins from green tea [46]. The eutectic mixture based on ChCl and glycerol showed the highest chemisorption capacity and selectivity for all the target molecules from the natural plants, proving an excellent extraction ability.

## 3. Biocompatibility of DESs

### 3.1. DNA Stability and Behavior in DESs

DNA plays an important role in the development of biosensors. Biosensors based on nucleic acids are developed towards the recognition of different organism strains through hybridization (genosensors) or exploit the ability of ssDNA to fold and bind specific target molecules (aptasensors) [47].

DNA is usually employed in aqueous solutions; however, its helix structure can be affected by non-physiological temperatures, pH values, and ionic strengths. In addition, degradation processes promoted by nucleases can lead to its chemical instability [48]. This factor can be a drawback in biotechnological processes based on nucleic acids. Furthermore, very small water volumes vaporize immediately under open-air conditions or at high enough temperatures; this can be a further limitation in the development of DNA-based nanotechnologies, where extremely small volumes are usually employed. Consequently, the use of solvents without these limitations could provide new possibilities in the development of DNA-based devices.

It has been reported that DESs based on ChCl guarantee long-term stability of biomolecules such as DNA and proteins [49]. Recently, Zhao et al. described that DNA is able to form a stable duplex in reline. This interaction between DESs and DNA comes from the electrostatic attraction between organic cations and the DNA phosphate backbone, accompanied by hydrophobic and polar interactions between eutectic liquids and the DNA major and minor grooves [50]. In the case of ChCl-based DESs, choline ions establish an interaction with atoms of all areas of DNA thanks to the creation of multiple hydrogen bond networks that are able to stabilize the DNA duplex more effectively than the sodium ions present in aqueous buffers. As to DNA stability, cations play a more important role than anions because cationic species are required to weaken the repulsive forces between phosphate groups of DNA strands, while anions only establish an interaction with the bases through hydrogen bonds [51].

**Figure 6:**
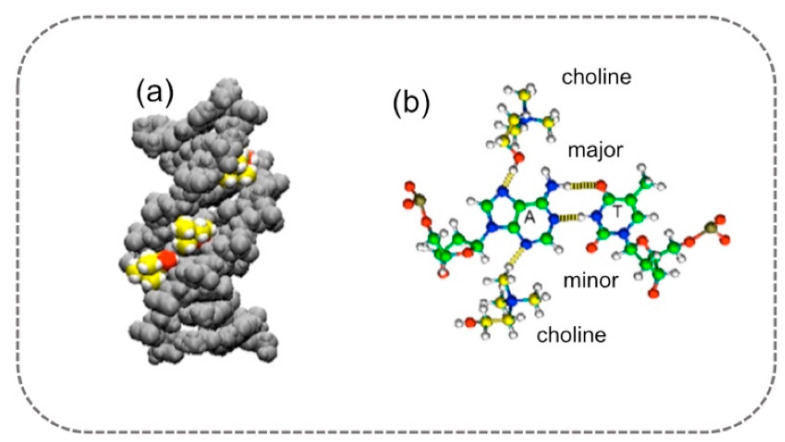
Molecular dynamics simulation of (**a**) choline ions solvating the minor groove of A–T base pairs of duplex DNA; carbon atoms of choline ions are represented in yellow, oxygen atoms of choline ions are represented in red, and hydrogen atoms are in white. (**b**) Atomic-scale representation of the DNA–choline bonds. Adapted with permission from Nakano, M.; Tateishi-Karimata, H.; Tanaka, S.; Sugimoto, N. Choline Ion Interactions with DNA Atoms Explain Unique Stabilization of A–T Base Pairs in DNA Duplexes: A Microscopic View. *J. Phys. Chem. B* **2014**, *118*, 379–389, doi:10.1021/jp406647b. Copyright 2014 American Chemical Society [51].

As shown in Figure 6, choline ions have a high affinity for the A–T base pair minor groove, which displays a smaller width and more electrostatically polar environment of this groove compared to the major groove. As can be noted, choline ions fit well into the minor groove of A–T base pairs, and as reported from simulations, this interaction persists over time. Furthermore, the multiple hydrogen bonds formed between choline ions and DNA atoms preserve the conformation of the A–T-rich DNA duplex.

Mamajanov et al. demonstrated that at least four distinct nucleic acid secondary structures can exist in DESs; they also noted that the anhydrous character and capacity to support natively folded DNA and protein structures make DESs appealing for the synthesis of biopolymers [52]. Pal et al. examined the interaction between reline and guanine-rich quadruplex thrombin-binding aptamer (TBA) at 300 K for different reline concentrations [53]. They studied the conformational behavior of the aptamer in reline using molecular dynamics simulations. The results showed that the structure and conformation of the sequence were more rigid than those in the lower reline concentration. The experimental findings confirmed the structural features of TBA in anhydrous and hydrated reline media. Overall, this study indicates that reline is suitable for long-term nucleic acid storage. Moreover, Pal et al. also reported that the hydrogen bonds between reline and the proto-oncogene c-kit G-quadruplex DNA are responsible for its high temperature stability [54]. The authors studied the conformational dynamics of c-kit oncogene promoter G-quadruplex DNA in reline in a temperature range of 300−500 K, exploiting a 10 μs molecular dynamics simulation. They demonstrated that reline is able to protect the c-kit G-quadruplex DNA, and it also decelerates the thermal denaturing process.

Lastly, Svigelj et al. reported that the use of a DES in the selection of aptamers allowed a faster enrichment of highly affine sequences [42]. The authors employed ethaline to select aptamers against gliadin, which is a poorly water-soluble fraction of gluten. The effect of ethaline on the selection is probably due to the ability of DESs to stabilize secondary DNA structures, combined with the high viscosity which may add further restriction during the selection of aptamers. The development of DNA-based biosensors in DESs is discussed in detail in Section 4.

### 3.2. Enzyme Activity in DESs

Advances in biocatalysis have been made by performing enzymatic bioreactions in partly or totally nonaqueous solvents. This aspect can be particularly interesting when working with hydrophobic substrates, since the presence of organic solvents could deactivate or denature the employed enzyme. In biotransformation, DESs can take part as solvents, cosolvents, or a second phase in water−DES mixtures. The most interesting aspect of employing DESs in biocatalytic reactions is that their presence can recreate an environment very similar to the intracellular one, playing a new role different to that of water and lipids [55].

**Scheme 1:**
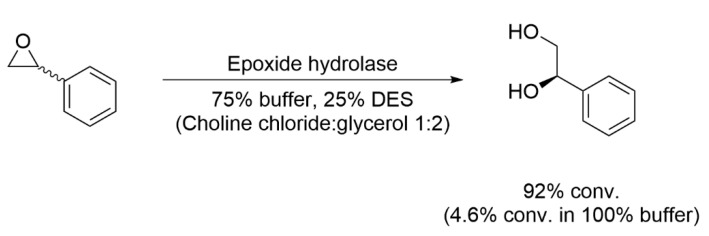
Scheme of epoxide hydrolase activity in DES.

For example, the use of DESs with hydrolases has been widely investigated [56]. Gorke et al. reported that the rate of hydrolysis of styrene oxide catalyzed by epoxide hydrolase increased dramatically in DES: the conversion was only 4.6% in buffer but increased to 92% upon addition of 25 vol% ChCl:glycerol, with unchanged enantioselectivity (Scheme 1) [57].

The use of DESs can also impede enzyme denaturation and deactivation, phenomena that are frequently observed in conventional organic solvents, as reported in some works with horseradish peroxidase (HRP) and hydrolases. In addition, DESs have a positive influence on HRP stability and enhance cytochrome C activity [58,59]. As a matter of fact, Ghobadi et al. reported an increased binding affinity of H_2_O_2_ to Bovine liver catalase when using a ChCl-based DES [60].

The effect of DESs on enzymes such as laccase is also an interesting aspect. Laccase is an oxidase that is usually employed in biosensors to detect phenolic compounds [61]. The sensitive element of this kind of sensor is the actual enzyme with its active center, which is able to catalyze electron transfer reactions without cofactors. The performance of enzyme biosensors can be influenced by pH, temperature, and the activity of the enzyme [62]. Laccase-catalyzed reactions have been studied in 16 different DES aqueous solutions. In their work, Toledo et al. described that while ChCl-based DESs led to a decrease in laccase activity, the substitution of the chloride anion in the cholinium salts with other anions significantly promoted the laccase activity [63]. Specifically, the DES choline dihydrogen phosphate:xylitol and all choline-dihydrogen-citrate-based DESs resulted in an improvement in the activity of laccase in oxidative reactions. Moreover, the laccase activity in these DES aqueous solutions was higher than that in aqueous solution of its individual components and in the buffer control, indicating a cooperative effect. Lastly, it was proved that water–DES mixtures can be employed as media for storing the enzyme with better performance than the classic buffer control at −80 °C, extending the activity up to 20 days.

## 4. Application of DESs in the Development of Biosensors

As stated in Section 2 and Section 3, the advantages obtained in the use of DESs for the synthesis of electrode materials and the manipulation of biomolecules can be fruitfully applied to biosensor development. Da Silva et al. proposed a biosensor with an innovative electrode structure based on the redox polymer poly(brilliant cresyl blue) (PBCB) electrodeposited in ethaline on a multiwalled carbon nanotube (MWCNT)-modified glassy carbon electrode (GCE) [64]. The authors optimized the electropolymerization in DES and performed the corresponding film characterization. Afterwards, they proposed its application in enzyme-based biosensors obtained by immobilizing glucose oxidase (GOx) or tyrosinase on PBCB Ethaline—HNO_3_/MWCNT/GCE. The authors compared three configurations, namely, GOx/PBCB Ethaline-HNO_3_/MWCNT/GCE, GOx/aqueous PBCB/MWCNT/GCE, and GOx/GCE, on which the same quantity of GOx was immobilized. The setup with PBCB deposited in DES showed the best sensitivity and a limit of detection of 2.9 mM for glucose determination by fixed potential amperometry. Moreover, the same approach was used to prepare tyrosinase biosensors. The Tyr/PBCB Ethaline-HNO_3_/MWCNT/GCE proved to be the best setup for the amperometric determination of catechol, as occurred for glucose with GOx.

Da Silva et al. also proposed an electrochemical enzyme inhibition biosensor for the pesticide dichlorvos based on choline oxidase (ChOx) immobilized on PBCB films electrodeposited in ethaline on a MWCNT-modified GCE [65]. Superior electrochemical performance of PBCB nanostructured films in ethaline was reported. In fact, ethaline, employed as a polymerization solvent for these films, led to smoother and more compact nanostructured surfaces compared to the ones obtained in aqueous media. This innovative platform presented excellent analytical parameters compared to other biosensors developed for monitoring traces of pesticides in different matrices.

**Figure 7:**
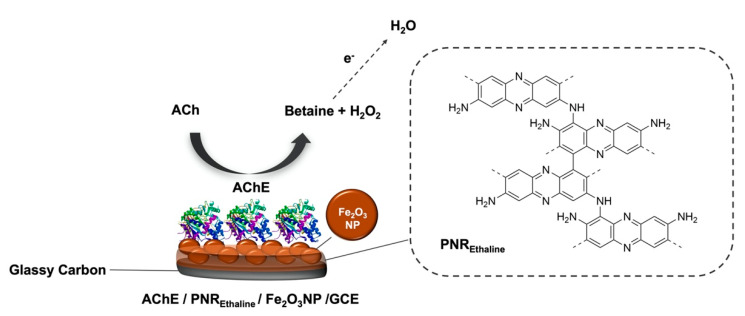
Schematic representation of the biosensor for acetylcholine detection. The glassy carbon electrode was modified with Fe_2_O_3_ nanoparticles; then the electropolymerization of neutral red in ethaline solution was performed, followed by immobilization of acetylcholinesterase (AChE).

More recently, another work from da Silva et al. described the development of an amperometric biosensor for the detection of acetylcholine (ACh) based on acetylcholinesterase (AChE). The enzyme was immobilized on poly(neutral red) (PNR) films generated by electropolymerization on Fe_2_O_3_ magnetic-nanoparticle-modified GCEs (Figure 7) [66]. Ethaline was employed for the electropolymerization of neutral red by potential cycling. Since eutectic solvents have low ionic strength, ethaline was added with acid dopants (HNO_3_, H_2_SO_4_, HCl, and HClO_4_). The composition of the ethaline–acid solutions influenced the morphology and, consequently, the electrochemical behavior of the polymer film. The biosensor was tested for the determination of ACh in synthetic urine and exhibited good selectivity, reproducibility, stability, and high selectivity, with rapid response and low limit of detection. The good results obtained for the analyte open the possibility to use this biosensor in clinical diagnosis.

**Figure 8:**
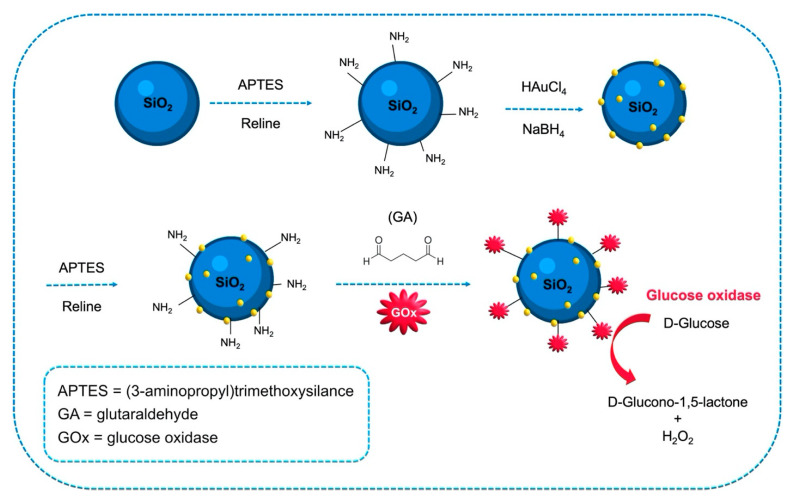
Scheme of the amine functionalization of SiO_2_ NPs (green) in DES, and the following immobilization of AuNPs (yellow) and of glucose oxide (GOx) on the nanoparticle surface. (Adapted from [67] with permission).

Kumar-Krishnan et al. demonstrated a simple and green synthetic approach based on the use of reline for the amine-surface functionalization of silicon dioxide and the integration of gold nanoparticles (AuNPs) for the preparation of a glucose biosensor [67]: the FAD-center of glucose oxidase (GOx) was covalently linked to the amine groups of the functionalized Au-SiO_2_ NPs using glutaraldehyde as bifunctional cross-linker (Figure 8). Thanks to the higher viscosity of reline in comparison to aqueous solvents, it was possible to obtain uniform surface functionalization with better stability and dispersion.

Afterwards, GCEs were modified with Au-SiO_2_NPs/GOx and employed for glucose quantitation. The biosensor showed direct electron transfer for the sensing of glucose with a sensitivity of 9.69 μA mM^−1^, wide linear range from 0.2 to 7 mM, and very good stability. This green DES-based synthetic procedure opens up the possibility of supporting different metal nanoparticles on SiO_2_ and, therefore, their subsequent use in the development of biosensors.

Two forms of nanocellulose obtained by exploiting a DES were applied by Ling et al. as platforms to enable the colorimetric detection of human neutrophil elastase (HNE) [68]. A DES based on ChCl and oxalic acid was employed for the formation of cotton cellulose nanocrystals (DCNCs), which were later used for the assembly of a protease sensor for HNE based on a peptide–cellulose conjugate. A comparison between the tetrapeptide–cellulose analog on DCNC and the analogous derivative of TEMPO-oxidized wood cellulose nanofibrils (WCNFs) was carried out. DCNCs proved to have a higher degree of substitution of HNE tetrapeptide and also a better sensitivity compared to WCNFs. X-ray diffraction (XRD) models showed the higher crystallinity and larger crystallite sizes of DCNCs. The sensitivity of this colorimetric biosensor sensor was less than 0.005 U/mL, reaching HNE levels that are usually found in human inflammatory diseases. Moreover, the authors stated that the cellulose derived from this innovative DES-mediated treatment could supply a new approach for the control of the nanocellulose crystal sizes, content of carboxyl groups, and surface area, which usually are decisive parameters for obtaining high-performing HNE sensors.

**Figure 9:**
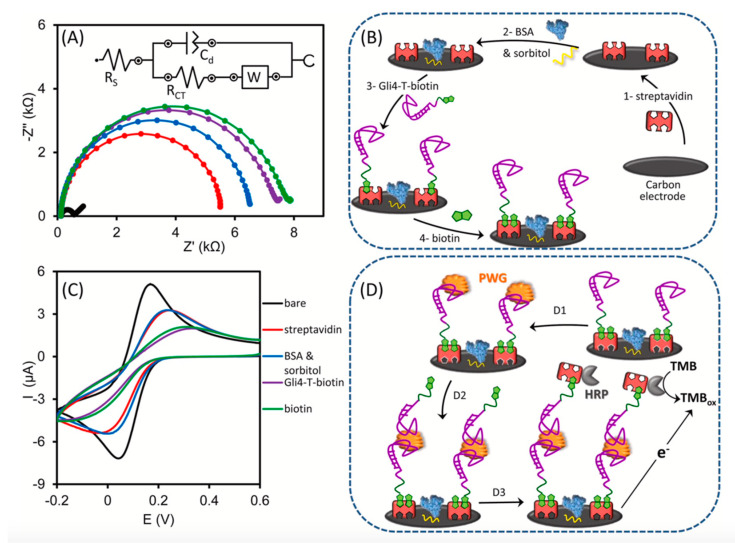
Sandwich biosensor based on a truncated aptamer capable of recognizing gliadin directly in a DES: (**A**) Nyquist plots of 0.5 mM [Fe(CN)_6_]^3−/4−^ after each modification step involved in the assembly of the biosensor, which are schematically represented in (**B**). (**C**) Voltammetric measurements recorded in KCl 0.1 M with 0.5 mM [Fe(CN)_6_]^3−^ after each modification step. (**D**) Steps involved in the sandwich assay; D1: interaction with the sample containing gliadin, D2: incubation with the second aptamer labelled with biotin, D3: incubation with streptavidin–HRP conjugate and chronoamperometric measurement of the oxidized tetramethylbenzidine enzymatically produced. (Reprinted with permission from [69]).

Svigelj et al. proposed a sandwich biosensor based on a truncated aptamer capable of recognizing gliadin directly in a DES [69]. The sensor used Gli4-T as the capture and signaling aptamer, one immobilized on the carbon working electrode and the other used to reveal the binding of the target protein after incubation with streptavidin–horseradish peroxidase (Figure 9). The biosensor showed a dynamic range between 1 and 100 μg/L and an intra-assay coefficient of variation of 11%. With this analytical performance it was possible to quantify 20 μg of gluten per kilogram of food when 1 g of food was extracted with 10 mL of ethaline. This aptamer-based sensor was directly deployed in ethaline, which was used for the extraction of gluten proteins from food. The optimization of the aptamer by truncation is a key step in the design. This sensor provides a new and faster way of analyzing residual levels of gluten in food, after extraction in DES, offering a promising tool to monitor and assess gluten in foods for highly sensitive celiac people.

**Figure 10:**
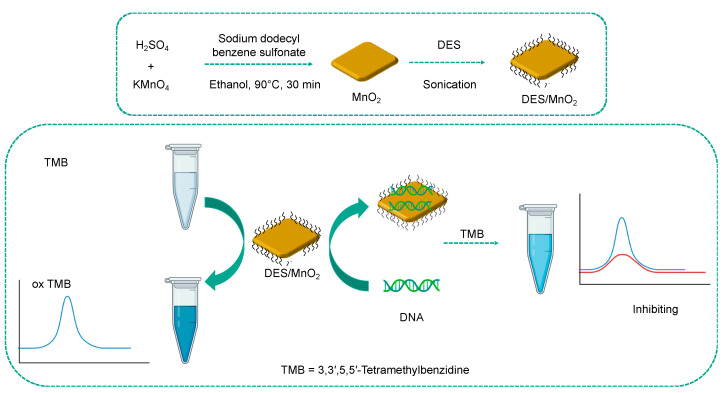
Preparation of DES/MnO_2_ and its application for the colorimetric detection of DNA. Adapted with permission from [70].

Finally, Chen et al. described a composite generated by the coupling of a DES and MnO_2_ nanosheets (DES/MnO_2_) [70]. This composite was shown to be able to oxidize 3,3′,5,5′-tetramethylbenzidine (TMB) to the oxidized blue product (oxTMB) which presents a maximum absorbance peak at 652 nm. The DNA molecules, which are typically negatively charged, were immobilized on the surface of the composite by hydrogen bonding interactions and electrostatic interactions, thanks to the presence of DES. The adsorption of DNA molecules caused the suppression of the oxidase-like activity of DES/MnO_2_ (Figure 10). The authors took advantage of this result to set up a colorimetric approach for the detection of DNA. The DES-based system showed high selectivity for DNA against several targets of biological nature; however, the presence of RNA in the sample can cause interference. The sensor was also applied in the analysis of real samples, such as bovine whole blood, demonstrating that this method could be easily used for the detection of DNA in complex matrices. This assay has a dynamic range of 10–100 μg mL^−1^ and a detection limit of 0.37 μg mL^−1^.

## 5. Conclusions and Future Perspectives

In this review, we collected recent progress in the use of DESs for the development of biosensors. Considering their high compatibility with DNA, enzymes, and MIPs, which are popular recognition elements, and their ability to stabilize nanomaterials, it is clear that the use of DESs can bring relevant advantages for electrode modifications; furthermore, these solvents can be exploited to develop biosensors with improved sensitivity and to enable poorly soluble molecules to be detected.

Finally, the use of DESs could allow enormous advances in the field of biotechnology: they could play a significant role in advancements in DNA-related technologies, enzyme-based procedures, and MIP production. In the light of all DESs’ features, including their easy preparation, biodegradability, high stability, and low volatility, the possibility of using DESs in biosensing implementations appears to be of unquestionable interest. Despite the great progress obtained in the last years, there are still many unexplored research routes in biosensing development using DESs.

In particular, it is necessary to increase the knowledge about the influence of DESs on the mechanisms of enzyme functioning and the interactions between aptamers and ligands; also, the strategies of electrode material modification with DESs should be further expanded to lead to high-level technological developments.

Moreover, DESs’ ability to cover a wide range of polarity opens up the possibility of creating analytical platforms for a series of molecules for which it has not been possible to develop an effective methodology so far because of solubility reasons. For instance, lately, DESs have been used as extraction solvents during the pre-concentration of pesticide residues in food and environmental samples, with subsequent instrumental analysis [71].

Considering also the biocompatibility of these media with cells, DNA, proteins, and enzymes, it is reasonable to think that they could play a relevant role in biosensing developments for biomedicine targets.

Finally, DESs are nontoxic and environmentally friendly, characteristics that make them appealing for applications as green media in the development of devices for the pharmaceutical, agrochemical, and food industries.

## Figures and Tables

**Table 1:** Physical parameters of some ChCl-based DESs.

DESs	Salt/HBD Molar Ratio	Viscosity ^a^ Pa·s	Conductivity ^a^ μS·cm^−1^	Density ^a^ g/cm^3^	T_f_/°C
ChCl:urea	1:2	-	-	1.25	12
ChCl:acetamide	1:2	0.127	2710	1.09	51
ChCl:glycerol	1:2	0.177	1647	1.19	−40
ChCl:1,4-butanediol	1:4	0.047	2430	1.04	-
ChCl:triethylene glycol	1:4	0.044	1858	1.12	−66
ChCl:xylitol	1:1	3.867	172.6	1.24	-
ChCl:D-sorbitol	1:1	13.736	63.3	1.28	-
ChCl:oxalic acid	1:1	0.089	2350	1.24	34
ChCl:levulinic acid	1:2	0.119	1422	1.13	-
ChCl:malonic acid	1:1	0.616	732	1.21	10
ChCl:malic acid	1:1	11.475	41.4	1.28	-
ChCl:citric acid	1:1	45.008	18.4	1.33	69
ChCl:tartaric acid	2:1	66.441	14.3	1.27	47

^a^ Values determined at 30 °C; T_f_ = freezing point.

## Data Availability

No new data were created or analyzed in this study. Data sharing is not applicable to this article.
